# Cytokinin biosynthesis in cyanobacteria: Insights for crop improvement

**DOI:** 10.3389/fgene.2022.933226

**Published:** 2022-09-09

**Authors:** Shashi Uniyal, Munni Bhandari, Preeti Singh, Rahul Kunwar Singh, Shree Prakash Tiwari

**Affiliations:** ^1^ Department of Microbiology, School of Life Sciences, H.N.B Garhwal University, Srinagar, Uttarakhand, India; ^2^ Department of Microbiology, V.B.S Purvanchal University, Jaunpur, Uttar Pradesh, India

**Keywords:** cyanobacteria, cytokinins, cytokinin biosynthesis, crop improvement, sustainable agriculture

## Abstract

Cytokinins, a type of phytohormones that induce division of cytoplasm, have considerable value in agriculture due to their influences on several physiological processes of plants such as morphogenesis, development of chloroplast, seed dormancy, leaf senescence, etc. Previously, it was assumed that plants obtain cytokinin from the soil produced by microbes as these hormones were first discovered in soil-inhabiting bacteria i.e., *Agrobacterium tumefaciens*. Later, the cytokinin biosynthesis gene, i.e., ipt gene, has been reported in plants too. Though plants synthesize cytokinins, several studies have reported that the exogenous application of cytokinins has numerous beneficial effects including the acceleration of plant growth and boosting economic yield. Cyanobacteria may be employed in the soil not only as the source of cytokinins but also as the source of other plant growth-promoting metabolites. These organisms biosynthesize the cytokinins using the enzyme isopentenyl transferases (IPTs) in a fashion similar to the plants; however, there are few differences in the biosynthesis mechanism of cytokinins in cyanobacteria and plants. Cytokinins are important for the establishment of interaction between plants and cyanobacteria as evidenced by gene knockout experiments. These hormones are also helpful in alleviating the adverse effects of abiotic stresses on plant development. Cyanobacterial supplements in the field result in the induction of adventitious roots and shoots on petiolar as well as internodal segments. The leaf, root, and stem explants of certain plants exhibited successful regeneration when treated with cyanobacterial extract/cell suspension. These successful regeneration practices mark the way of cyanobacterial deployment in the field as a great move toward the goal of sustainable agriculture.

## 1 Introduction

Cytokinins (CKs) are N^6^-substituted adenine derivative phytohormones responsible for nutrient uptake, cell division, embryonic development, and the overall growth of plants (Mishra et al., 2022). The word “cytokinin” has been derived from the role of these compounds in cytokinesis i.e., cell division. The cytokinins were discovered in the 1950s by Skoog et al., and play critical roles in the interaction of plants with different biotic and abiotic factors (Han et al., 2022). The first cytokinin, kinetin (6-furfural-aminopurine), was discovered as a degradation product of herring sperm DNA (Le Bris, 2017). However, the first natural cytokinin, zeatin, was purified from immature maize kernels. These hormones are abundantly found in shoot and root apexes, as well as in immature seeds. CKs are functionally active in plants at every minute i.e., micro to nano molar concentration (Žižková et al., 2017). The side chains available in the cytokinin hormones have a great influence on its biological activity depending on its configuration and conformation.

Based on the available side chains, isoprenoid and aromatic cytokinins are the two broad groups of CKs. Isoprenoid CKs are the more abundant category as compared to the aromatic ones (Raspor et al., 2020). Isoprenoid CKs include N^6^-(∆^2^-isopentenyl adenine (iP), dihydrozeatin (DHZ), trans- as well as cis-zeatin, while aromatic CKs include N^6^-Benzyladenine (BA) and its hydroxyl derivatives such as ortho- and meta-topolin (Žižková et al., 2017). Natural CKs undergo a certain kind of modifications in which amino acids or sugar moieties like alanine, glucose, or ribose get attached to the basic cytokinin structure (Hošek et al., 2020). In plants, cytokinin occurs as nucleotides, nucleosides (ribosides), glycosides (O- and N-glycosides) and free bases depending on the molecule conjugated to the basic cytokinin structure (Ashihara et al., 2020). For example, when a ribose sugar gets attached to the purine ring at the N^9^ position, it is termed as a riboside. If a phosphate group is also present along with the ribose sugar, it is called a ribotide (Kalra and Bhatla, 2018). Similarly, O- and N-glycosides are formed by the addition of sugar moiety, commonly glucose, at O (at hydroxyl group of N^6^ side chain) and N (N^3,^ N^7^ and N^9^) sites, respectively (Pokorna et al., 2020). Based on the physiological activity, CKs can be grouped under two different categories. The first category comprises of highly active forms and includes free bases, nucleosides, and nucleotides, whereas another category encompasses moderately active CK-N and CK-O glycosides (Žižková et al., 2017). Ribosides are the form with lower activities while free bases are forms with higher activities. The higher abundance of inactive forms as compared to the active forms suggests that the enzymes involved in the synthesis, modification, and degradation of cytokinins are regulated in a controlled and coordinated manner (Kroll and Brenner, 2020).

The biosynthesis of cytokinins is catalyzed by the enzyme isopentenyl transferase encoded by the *ipt* gene. The gene was initially discovered in the soil-inhabiting bacterium i.e., *Agrobacterium tumefaciens* (Nester et al., 1984). Plants were known to obtain cytokinin from soil. Later, the gene was discovered in plants as well which led to the consideration that plants are able to produce cytokinin (Kakimoto, 2001). The fact has raised a question that if plants themselves are capable of producing cytokinin, what is role of cytokinin-producing microorganisms in the soil? Cytokinin-producing microbes further enhance the level of cytokinin in plants and soil as well (Akhtar et al., 2020).

Cytokinins are known to alleviate the damage engendered by different abiotic stresses (Bryksová et al., 2020) and it also has wide application in plant tissue culture due to its stimulatory impact over the regeneration of plants (Grzegorczyk-Karolak et al., 2021). Further genetic manipulations in the cytokinin metabolism genes can lead to enhancements in crop growth parameters (Liu et al., 2020). Due to its tremendous applications in agriculture and possibilities for improvement in its metabolic enzyme pathways to achieve even better results, cytokinin has become a subject requiring more concern and attention. Hence, the present article attempts to discuss the different applications of cytokinins in agriculture, cyanobacteria as exogenous sources of cytokinins, cytokinin metabolism in cyanobacteria, and genetic manipulation strategies to improve its production.

## 2 Applications of cytokinins in agriculture

As mentioned in the earlier section, cytokinins have a wide range of applications in agriculture as they promote overall plant growth. These hormones along with auxins are responsible for maintaining root and shoot growth. Also, an establishment of a balance between cytokinins and salicylic acid is very critical during the early development of the plant i.e., during the initial stage of seed germination and root elongation (Toribio et al., 2020). The specific application of cytokinins in agriculture is discussed below.

### 2.1 Regulation of leaf development

Leaves are the major organ of a plant that helps in perceiving light and responding to external environmental conditions. Leaves and other aerial organs of plants are generated from three functional zones, i.e., CZ (Central Zone), RB (Rib Zone), and PZ (Peripheral Zone) of the shoot apical meristem (SAM) (Wu et al., 2021). SAM is a highly organized tissue located at the shoot apex and consists of pluripotent cells (Armenta-Medina and Gillmor, 2019). Cytokinins are considered to be important for the proper care of SAM. The size as well as activities of SAM are controlled by cytokinins as a decline in cytokinin level, either by mutation in the *ipt* gene or by an overexpression of the *CKX* gene, may lead to the reduction in the activity and size of SAM (Wu et al., 2021). In addition, cytokinins have a key contribution in the organization of the leaf layout. Marginal blastozone (MB), a region at the leaf margin, gives rise to lobes or leaflets in a leaf (Wu et al., 2021). For this, the stem cell or meristematic activity requires to be maintained for a longer duration for the formation of leaf and this is achieved through cytokinins (Wu et al., 2021). Also, the structure of a developing leaf can be altered by modifying the endogenous cytokinin level (Shamsi et al., 2019). Thus, it can be summarized that cytokinins play critical roles such as the regulation and modulation of genes as well as signaling during leaf development (Wu et al., 2021). Apart from this, CKs are helpful in maintaining leaf health as these hormones not only postpone the leaf senescence but also increase the size of stomatal apertures and rate of transpiration in numerous plants (Guo et al., 2021).

### 2.2 Roles of cytokinins in seed enhancement

Seed enhancement is a process or a technique through which the germination efficiency of a seed is improved and growth of the seedling is enhanced. Cytokinins play roles in seed enhancement as the cytokinin-primed wheat seeds are known to have better germination, growth, and yield under salt stress condition (Rhaman et al., 2020). A study to evaluate the impact of seed priming with two different cytokinins, kinetin and benzylaminopurine (BAP), was conducted by Iqbal et al. (2006). They observed that the germination rate and early seedling growth rate of both, salt-intolerant and salt-tolerant cultivars under salt stress were enhanced after priming with kinetin.

### 2.3 Regulation of plant response to stress

Cytokinins not only play their role in normal growth, but also help in the redistribution of essential compounds like nucleic acids, inorganic salts, and hormones throughout the plant body (Ullah et al., 2018). This re-distribution inhibits the degradation of chlorophyll, proteins, and nucleic acids and thus restricts the senescence of plants. Further, it can be inferred from recent research studies that cytokinin has the potential to ameliorate the impairments caused by different stress conditions, such as heat, cold, drought, and salt (Prerostova et al., 2018). Under a heat stress situation, the production of reactive oxygen species (ROS) is geared up (Hu et al., 2020). Cytokinins mitigate the effect of ROS and impart the heat-tolerance ability to plants by stimulation of the antioxidant system as well as upregulation of heat shock response proteins (Li et al., 2021). Similarly, the cold-tolerant capacity of plants can be enhanced by enhancing the cytokinin level either exogenously or endogenously (Liu et al., 2020).

Crop production is frequently impacted by a variety of abiotic challenges; hence, crop protection against abiotic stress is critical to their survival. Water shortage, heat stress, drought, high and low salt concentration in soil, as well as high and low temperature are example of abiotic stressors (Ronga et al., 2018). These stressors induce phenotypic, genotypic, and metabolic variations in plants that have a negative impact on their growth and productivity (Ronga et al., 2018). Cyanobacteria might be considered a prospective source of new biostimulants as these organisms may help to ameliorate the negative consequences of abiotic stress and promote plant growth by producing a variety of chemicals (Santini et al., 2021). Auxins, cytokinins, betaines, amino acids, vitamins, and polyamines are such chemicals produced by cyanobacteria with very low effective concentrations (Ronga et al., 2019). Among these chemicals, CKs are significant regulators of plant activities under harsh ecological situations (Castander-Olarieta et al., 2021). Thus, cyanobacteria, such as *Nostoc entophytum* and cyanobacterial filtrates, containing high levels of cytokinins and auxins (IAA), could be a new avenue for applying exogenous phytohormones in agriculture (Ronga et al., 2019; Kollmen and Strieth, 2022).

Exogenous and endogenous cytokinins have different effects on stress tolerance by plants (Liu et al., 2020). Exogenously supplied cytokinin can not only improve salt tolerance, but can also lead to a phenotypic variety more vulnerable to salt treatment (Quamruzzaman et al., 2021). All drought-stressed plants showed growth inhibition, which was linked to an enhanced level of abscisic acid and a reduced levels of auxins and active cytokinins (Prerostova et al., 2020).

Apart from its role in drought resistance, CKs also play a role in temperature detection and heat signaling in *Arabidopsis* (Castander-Olarieta et al., 2021). The impact of exogenous CK administration on modulating stress tolerance has been investigated with the help of a wide range of hormone-treatment options. Cyanobacterium, *Synechocystis* sp., can tolerate salt levels up to 1.2 M NaCl due to cytokinin production (Yang et al., 2020). On cytokinin-supplemented media bean plants, sprouting potato tubers and wheat seedlings showed better salt tolerance (Naqvi et al., 1982; Abdullah and Ahmad, 1990). Spraying with cytokinin before a drought boosted the capacity of bean plants to tolerate stress but had a negative impact on maize and sugar beet (Zwack and Rashotte, 2015). More so, *Arabidopsis* growing on a cytokinin-supplemented medium had resulted in a higher yield. Cytokinin-supplemented plants had shown a higher survival rate than non-supplemented plants in conditions of cold or dehydration stress also (Zwack and Rashotte, 2015). Another example is heat tolerance in tobacco and radiata pine plants, as well as improved osmotic defense, recovery, regulated photosynthesis, and antioxidants (Prerostova et al., 2020; Castander-Olarieta et al., 2021).

Furthermore, CKs has also been shown to have a favorable influence on photosynthesis. Benzyladenine (BA) is a naturally occurring cytokinin that increases CO_2_ fixation, resulting in increased sugar production and preserving the chloroplast under stress conditions. CKs may potentially act as non-enzymatic ROS scavengers. For example, zeatin riboside is known to protect the viability of seeds by scavenging the superoxide anions. These findings suggest that a variety of factors such as geographical, temporal, and environmental factors determine how cytokinin therapy affects stress signaling.

### 2.4 Impact of cytokinins on root nodulation

Root nodules in leguminous plants are induced by rhizobia infection and controlled by nodulin gene expressions. Based on the expression timing, the nodulin genes can be categorized into early and late nodulin genes. Infection and nodule organogenesis are the concern of early nodulin genes while the nodule function, maintenance of the oxygen level by leghaemoglobin, etc., are regulated by the products of late nodulin genes (Lebedeva et al., 2021). Cytokinin is responsible for inducing the expression of early nodulin genes in legumes, cortical cell division, and ultimately, the formation of a nodulin-like structure (Yang et al., 2022). Mens et al. (2018) studied the role of cytokinin in the nodulation of soybean plants. They concluded that, cytokinin plays a critical role in initiating cortical cell division and thereby in expression of early nodulation transcription factors. Furthermore, nodule development was favored by an exogenous application of a low amount of cytokinin (0.1 µM). However, the nodulation can be reduced by the high concentration of cytokinin due to its toxic effects. They also observed that *ipt* genes are regulated in response to rhizobial inoculation (Mens et al., 2018).

### 2.5 Role of cytokinins in the interaction between plants and pathogenic microbes

Nowadays, scientists across the globe are focusing on the mechanism of interaction between the plant and phytohormones and among the hormones themselves (Kieber and Schaller, 2018). The interactions of phytohormones with each other are responsible for the regulation of the signaling pathways involved in metabolism which in turn affects the final outcome of the plant’s response to different environmental conditions and plant development (Lubyanova et al., 2021). This cross-talk between hormones involved in signaling is important for a plant to respond to the stress conditions (Yang et al., 2019). There are various synergistic and antagonistic interactions among the hormones which determine the overall behavior of a plant under different conditions. The interaction between phytohormones, JA (jasmonic acid), SA (salicylic acid), and ET (ethylene), is the key to the defense response of plants. The SA defense of plants is enhanced by cytokinin and thus cytokinin and salicylic acid exhibit a positive and beneficial interaction. A high level of cytokinin can modulate the SA signaling by triggering the SA-related gene expression involved in plant defense, thus, providing an enhanced protection to the plant (Lubyanova., 2021; Asif et al., 2022).

### 2.6 Role of cytokinins in chloroplast development

The controlled and coordinated regulation of the transcription of genes encoded by the chloroplast and nuclear genomes is the foremost requirement for the development and functionality of chloroplast. Cytokinins are known to be the major factor responsible for the expressions of genes encoding plastids and other related proteins. It is well known that CKs regulate the development of chloroplasts under various environmental conditions during different stages of plant development (Andreeva et al., 2020). These molecules regulate the expression of gene-encoding protochlorophyllide oxidoreductase enzyme having a critical role in chlorophyll synthesis. These hormones are also responsible for the synthesis of electron transport chain proteins in the thylakoid membrane (Toribio et al., 2020; Santini et al., 2021).

## 3 Exogenous sources of cytokinin

Cytokinins may be synthesized in a laboratory which has certain beneficial impacts. The exogenous application of synthetic cytokinins can also enhance plant growth parameters. It can improve the antioxidant enzyme activity and thus reduce the oxidative stress. Synthetic cytokinins such as Thidiazuron (TDZ) and CPPU N-(2-chloro-4-pyridyl)-N'phenylurea (CPPU), that can activate cytokinin receptors are known to improve chlorophyll *b* content and can be used as a cotton defoliant (van Voorthuizen et al., 2021).

The use of synthetic cytokinin, TDZ (0.25 mg/L) has been reported to adversely affect the shoot development in *Hibiscus acetosella* including poor shoot elongation and distortion. In case of the *Acer saccharinum* plant, a high concentration (1.0 mg/L) of TDZ caused fascination, i.e., formation of flat shoots resembling multiple shoots fused together and stunting and drop of leaves were observed (Trigiano and Gray., 2016). In another study, the suppression of shoot elongation was observed in the *Liquidambar* plant in response to TDZ. Another study observed the hyperhydricity of shoot tissues in the *Juglans nigra* plant when exposed to high concentrations of TDZ. In addition, the plants exhibited thicker stems, shorter internodes, as well as elongated, deformed, and brittle leaves, with an appearance of being soaked and translucent (Trigiano and Gray, 2016). More so, TDZ stimulates the establishment of the abscission zones and leaf abscission, increase in ROS levels, destruction of net photosynthesis, and a substantial decrease in transpiration and stomatal conductance by increasing the activity of cell wall-degrading enzymes and the ethylene content (Jin et al., 2020). On the other hand, the application of CPPU may cause a slight delay in the maturity of plants and ripening of fruits (Fujisawa et al., 2018).

In addition to synthetic sources, there are several natural exogenous sources of cytokinins such as insects, plant pathogenic bacteria, fungi, amoeba, and cyanobacteria (Frébortová and Frébort, 2021). Phytophagous insects are reported to produce cytokinin as evinced by the higher cytokinin content in the body of different insects as compared to plants. Cytokinin production by an insect aims to manipulate the plant defense response against herbivory and also to modify the distribution of nutrients at the site of feeding in a leaf (Andreas et al., 2020). Plant pathogenic microbes produce cytokinins as a virulence factor. For example, *Agrobacterium tumefaciens*, transfers T-DNA (Transfer- DNA, which is a section of the Ti plasmid, present in the bacteria and is responsible for inducing diseases in plants) containing the *ipt* gene into the host cell during infection. As this T-DNA gets integrated into the host genome, it leads to an overproduction of cytokinins and along with increased auxin content results in tumorous cell proliferation. Thus, the bacterium is responsible for crown gall disease. Similarly, *Pseudomonas syringae* and *R. fascians* also cause diseases in plants (Schmülling, 2013). Fungus *Claviceps purpurea*, amoeba *Dictyostellium discoideum,* have also shown the presence of adenylate IPTs (Frébortová et al., 2017).

In addition to plant pathogens, few human pathogens are also known to produce cytokinins. Earlier, the significance of cytokinin production by human pathogens was unknown and a challenge for researchers. Samanovic et al. (2018) demonstrated that the production of cytokinin by a human pathogen, *Mycobacterium tuberculosis*, is necessary to instigate changes in its transcription and physiology. Thus, cytokinin is used as a communication signal by pathogenic microorganisms. Though, these organisms are able to produce cytokinins, their application in agriculture is restricted due to their adverse effects on plants as well as in human health.

Although there are a number of external sources of cytokinin, cyanobacteria may be the most preferred source as these organisms have the ability to impart several other benefits to plants as well. The details of these benefits are given in a later section. Thus, cyanobacteria may be deployed in agricultural fields as biofertilizers leading to the effective enhancement in growth and yield of crops in a sustainable way (Žižková et al., 2017).

## 4 Cytokinin production by cyanobacteria

Cytokinins are crucial phytohormones responsible for stimulating the interaction between plants and cyanobacteria. There are many experimental evidences that support the cytokinin production by cyanobacteria and its further role in crop improvement. The cyanobacteria such as *Nostoc sp.* PCC7120, *Anabaena variabilis* ATCC 29413, *Microcystis aeruginosa* NIES-843, *Oscillatoria*, *Phormidium,* and *Chroococcidiopsis sp.* are known to produce cytokinins. Table 1 shows the different methods of cytokinin detection in cyanobacterial extract/cell suspension/dried biomass. These hormones are responsible for the induction of shoots as well as adventitious roots at petiolar and internodal segments (Zhao et al., 2021). Thus, cytokinins produced from cyanobacteria can be used for the *in vitro* regeneration of plants (Toribio et al., 2021). Toribio et al. (2020) observed an enhanced radicle and aerial growth of cucumber seedlings after cyanobacterial inoculation with *Trichormus* SAB- M304 and *Nostoc* SAB-M251, due to the cytokinin secretion by these organisms. Frébortová et al. (2017) have studied the effect of light on cytokinin production by *Nostoc* sp. and observed the enhanced production of cytokinins under dark conditions. A microscopic analysis evinced that the cyanobacterium, *Calothrix ghosei*, is capable of establishing a relationship with wheat plant roots by penetrating them and stimulating their growth (Karthikeyan et al., 2009).

**TABLE 1:** The techniques used for the detection of cytokinins in different cyanobacterial species.

Cyanobacteria	Process used for sample preparation	Technique used for quantification of cytokinin	References
*Arthronema africanum*	Cation exchange resin and paper chromatography	GC-MS	Stirk et al. (1999)
*Calothrix* sp	Ultrasonication	Soybean Callus Bioassay	Stirk et al. (2002)
*Chroococcidiopsis*	Homogenization, sonication	UPLC–ESI–MS/MS	Hussain et al. (2010)
*Chroococcus, Nostoc*	Homogenization	HPLC-MS/MS, UHPLC-MS/MS	Žižková et al. (2017)
*Nostoc* SAB-M251, Trichormus SAB-M304, Nostoc SAB-M612	Sonication	Immunodiagnostic test	Toribio et al. (2020)
*Nostoc* sp. HK-01	Sonication	HPLC-ESI-MS/MS	Kimura et al. (2020)
*Anabaena oryzae*, *Nostoc entophytum*	Homogenization	Gas liquid chromatography	EL-Zawawy et al. (2021)

Cytokinins are the primary hormones that initiate the cyanobacterial colonization in plant roots as evidenced by a gene knockout study. In this study, the *ipt* gene knockout of cyanobacterium, *Nostoc* sp., was created. The knocked-out organism was unable to colonize the plant root. The hypothesis behind this knockout study was that cytokinin production by the cyanobacterium would be ceased in the absence of the *ipt* gene as it codes for isopentenyl transferase, a biocatalyst critical for cytokinin biosynthesis. Furthermore, the absence of cytokinin would also reduce the root colonization potential of the organism (Lee and Ryu., 2021).

It has been experimentally proved that cyanobacteria can modify the endogenous phytohormone levels in an inoculated plant as observed in wheat. An *in vitro* study conducted by Hussain and Hasnain 2011, concluded that cyanobacterial inoculation led to the endogenous cytokinin and IAA accumulation (Santini et al., 2021). Table 2 shows examples of crops which exhibited resistance to abiotic stresses and enhanced yields when treated with cyanobacterial extracts/cell suspension. However, more research is needed to study the effects of cyanobacterial inoculation on various other crops.

**TABLE 2:** Physiological effects of cyanobacteria on different crops exposed to abiotic stress.

Cyanobacteria	Forms used		Crop	Relevant response in higher plants	Physiological impacts in higher plants	Responsible metabolites	References
*Scytonema hofmanii*	Extracellular products		Oryza sativa	Tolerance to salt stress	Hormone homeostasis	Cytokinin	Rodriguez et al. (2006)
*Spirulina maxima*	Water extract		Triticum	Salinity tolerance	Antioxidant activity, stimulate protein content	Carotenoid tocopherol, phenolic, and protein	Abd El-Baky et al. (2010)
*Oscillatoria acuta*, *Plectonema boryanum*	Cell suspension		Oryza sativa	Stress tolerance against abiotic stress	Increases enzyme activity of peroxidase, antioxidant activity	Phenylpropanoids and flavonoids	Singh et al. (2011)
*Phormidium tenue*	Crude extract		Caragana korshinskii	Increases shrub performance in crusted desert parts	Acts as a biofertilizer, seed germination, increase carbohydrate contents and photosynthetic activity	Polysaccharides	Xu et al. (2013)
*Calothrix elenkinii*, *Anabaena laxa*	Thermotolerant bacteria		*Foeniculum vulgare*, *Coriandrum sativum*, *Cuminum cyminum*	_	Increases the enzyme activity of peroxidase, antioxidant activity, plant growth promotion	-	Kumar et al. (2013)
*Microcystis aeruginosa* MKR 0105 and *Anabaena* sp. PCC 7120	Biojodis and Cyanobacteria		*Zea mays*	Tolerance to thermal stress	Antioxidant activity tolerance to thermal stress	-	Piotrowski et al. (2016)
*Nostoc muscorum*, *Anabaena oryzae*	Algal extracts		*Phaseolus vulgaris*	Tolerance to cold and drought stress and nitrogen deficiency	Enhanced growth and photosynthesis	-	Setta et al. (2018)
*Spirulina platensis*	Exogenously applied cynobacteria		*Vicia faba*	Salt tolerance	Increase photosynthetic activity and protein content	Carotenoids	Selem, (2019)
*pirulina platensis*	Cell suspension		*Zea mays*	Tolerance to salt stress	Tolerance to cadmium toxicity	-	Seifikalhor et al. (2020)
*Arthrospira platensis*	Cell hydrolyzate		*Petunia xhybrida*	Tolerance to salt stress	Increased the K+/Na + relationship and Stimulates shoot and bud formation	-	Bayona-Marcillo et al. (2020)
*Nostoc piscinale*		Cyanobacterium-based biostimulant	*Zea mays* (SY Zephir hybrid)	Stress tolerance	Faster vegetative growth and higher chlorophyll content, higher grain yield	-	Ördög et al. (2021)
*Roholtiella* sp		Foliar extract	*Capsicum annuum*	Tolerating salt stress	Antioxidants activities and accumulation of proline, shoot length increased, fresh and dry weights chlorophyll a and b increase	Proline	Bello et al. (2021)
*Leptolyngbya*-1267		Sonicated extracts	*Solanum lycopersicum*	Plant protects against biotic stresses	Biopesticidal effect, mitigation of bacterial canker, promote plant growth, root development	Exo-metabolites	Toribio et al. (2021)

## 5 Cytokinins in plant tissue culture/explant regeneration

Plant tissue culture is currently recognized as an important tool of plant biotechnology, since it provides a fresh way to plant production, propagation, conservation and manipulation. Plant nutrition and morphogenesis are increasingly being studied using *in vitro* cell and tissue culture techniques. These methods are based on three primary principles: 1) isolation of the explant from its natural habitat, 2) use of aseptic cultivation conditions to keep these isolates alive in controlled conditions, and 3) *in vitro* maintenance of the physical and chemical environments. To manage the tissue culture environment, a variety of synthetic compounds are used. The phytohormones, such as cytokinins and auxins produced by cyanobacteria, may be used as a culture media supplement to enhance culture growth and shoot regeneration. This is evident from the study of a researcher, who recorded the advantageous effects of adding cyanobacterial biomass to culture media on the development of beet root and pea explants. Similarly, the extract of the cyanobacterium, *Plectonema* sp., when added in the culture medium, stimulated somatic embryogenesis and organogenesis in sandalwood and paddy (Singh, 2014). Moreover, cyanobacterial spent media have also been found effective in generating *in vitro* callus from explants of *Stevia rebaudiana* Bertoni (Blinstrubienė et al., 2020).

## 6 Metabolism and signal transduction of cytokinins

**FIGURE 1:**
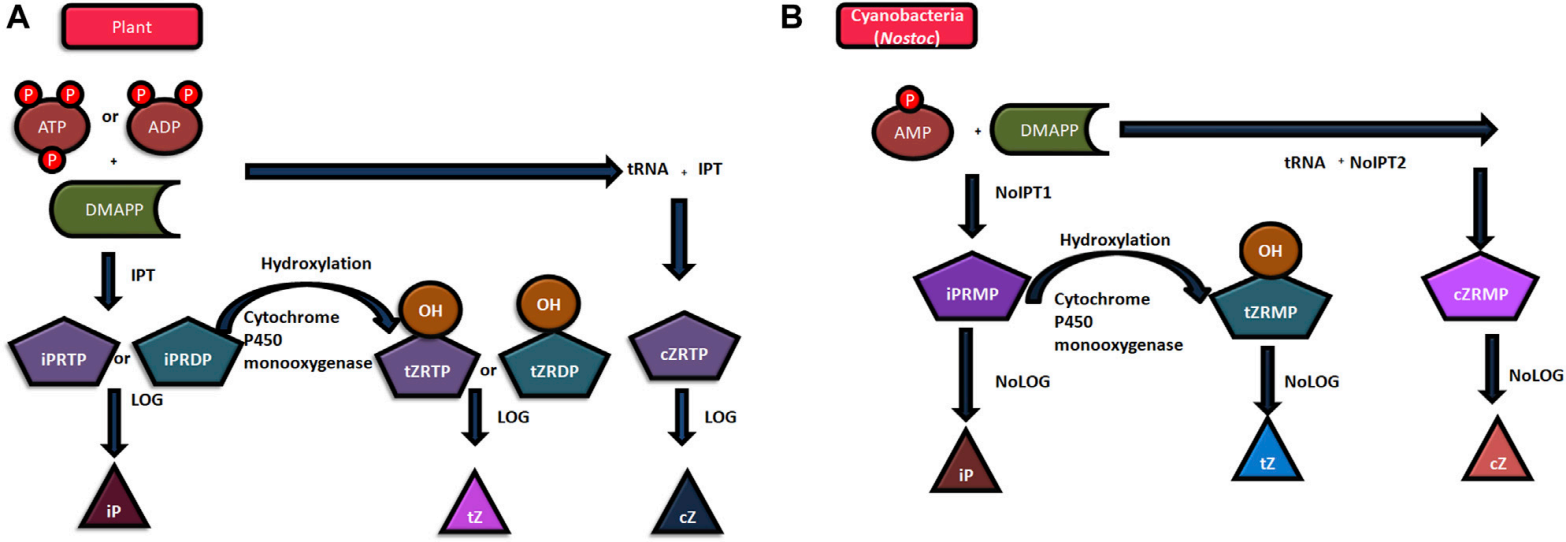
Mechanism of cytokinin synthesis **(A)** in plants, and **(B)** in Nostoc sp. PCC 7120. (Abbreviations: ATP- Adenosine triphosphate, ADP- Adenosine diphosphate, AMP- Adenosine monophosphate, DMAPP- dimethylallyl pyrophosphate; iPRMP- isopentenyladenosine-5- monophosphate; iPRDP- isopentenyladenosine-5- diphosphate, iPRTP- isopentenyladenosine-5- triphosphate tZRMP- trans-zeatin riboside 5′-monophosphate; tZRDP- trans-zeatin riboside 5′-diphosphate; tZRTP- trans-zeatin riboside 5′-triphosphate; cZRMP- cis-zeatin riboside 5′-monophosphate; cZRTP- cis-zeatin riboside 5′-triphosphate; iP- N6-(Δ2-isopentenyl) adenine; tZ-trans-zeatin; cZ-cis-zeatin, LOG- LONELY GUY; HMBPP- 4-hydroxy-3-methyl-but-2-enyl pyrophosphate).

The biosynthesis and degradation of cytokinin regulate its endogenous levels within the cells. The biosynthesis of isoprenoid cytokinin involves the handover of the isopentenyl chain from DMAPP (Dimethylallyl pyrophosphate) or HMBPP (4-hydroxy-3-methyl-but-2-enyl pyrophosphate) to AMP (bacteria), ADP, or ATP (plants) in the very first step to form isopentenyl -AMP, -ADP, -ATP, respectively. This reaction is catalyzed by the enzyme isopentenyl transferases (IPTs) of two different categories. The first one is adenylate IPT that catalyzes the transfer of the isopentenyl chain to the N^6^ amino group of free AMP, ADP, or ATP. The second one, tRNA- IPT, catalyzes its transfer to tRNA-bound AMP (Frébortová et al., 2017). The genes for both, adenylate IPT (*NoIPT1*) and tRNA-IPT (*NoIPT2*) have been identified in *Nostoc* sp. PCC7120 (Frébortová et al., 2017). Several researchers have concluded that there is much similarity between isopentenyl transferases (IPTs) in cyanobacteria and the same in plants (Žižková et al., 2017). However, the preferred substrate for plant IPT is ATP or ADP while AMP is the preferred substrate for cyanobacterial IPT. Furthermore, a majority of the cytokinin synthesis in plants is catalyzed by adenylate IPT, whereas tRNA IPT is the enzyme catalyzing most of the cytokinin synthesis in cyanobacteria. Figures 1A, B show the process of cytokinin synthesis in plants and cyanobacteria. Furthermore, the isopentenyl AMP, -ADP, and -ATP get converted to ribosides cytokinins, like zeatin, by hydroxylation of the isopentenyl side chain using the enzyme cytochrome P450 monooxygenase. The CKs, ribosides 5′monophosphates, are converted to free base CKs by the enzyme phosphoribohydrolase (Lonely guy; LOG). The interconversion of one form of cytokinin to another form continues so as to maintain and regulate the level of active compounds. Conjugates are formed by O and N glycosylations in the cytokinin (Frébortová and Frébort, 2021; Wu et al., 2021).

Although bacterial IPT uses both DMAPP and HMBPP as isopentenyl chain donors, in case of cyanobacteria, *Nostoc sp*. PCC 7120, the IPT enzyme (NoIPT1) was found to be more active when DMAPP was used as a donor. Since the reaction rate was very slow while using HMBPP as a side chain donor, further biochemical characterization in this case could not be done. Thus, it can be concluded that the cyanobacterial IPT resembles more to a plant’s IPT. The difference of IPT (plant and bacterial IPTs) in regard of the substrate may be due to different protein structures (Frébortová and Frébort, 2021; Wu et al., 2021).

Cytokinins can be inactivated or degraded through an enzyme known as cytokinin dehydrogenase (CKX) (Zhang et al., 2021; Mandal et al., 2022) which catalyzes an irreversible, oxidative cleavage of the cytokinin side-chain, which regulates the concentration of active cytokinins in plants. Multiple CKX proteins are found in terrestrial plants, each with its own tissue and subcellular localization as well as substrate specificity. Frébortová et al. (2015) carried out an investigation to explain the functionality of enzyme NoIPT1 along with NoCKX1 in *Nostoc* sp. The organism, *Nostoc sp.* was selected for the study so as to have a better understanding regarding cytokinin metabolism in cyanobacteria since it is not indulged in any kind of pathogenic activity in plants (Frébortová et al., 2015). The genes, *NoIPT1* and *NoCKX1* were isolated from *Nostoc sp.* and transformed in *E. coli* for the expression of proteins. The study observed that out of the two proteins i.e., NoIPT1 and NoCKX, only NoIPT was functional and had the characteristics of cytokinin dehydrogenase as found in plants. However, the *NoCKX* gene was not functional suggesting that the cyanobacterium was unable to perform cytokinin degradation through the CKX pathway (Frébortová et al., 2015).

Various efforts have been made to determine the signal transduction mechanism of cytokinins. As cytokinins are critical phytohormones, the model plant *Arabidopsis* was selected for studying the cytokinin signaling pathway. Cytokinins are upregulated by the plant cell through the interaction with the CHASE (cyclase/histidine kinase-associated sensing extracellular) domain of CRE1 (cytokinin receptor). The interaction of cytokinin to CRE1 leads to its autophosphorylation as it has a histidine kinase activity. After phosphorylation, it transfers its phosphate group to AHP (Arabidopsis Histidine Phosphotransfer) belonging to a histidine-containing phosphotransfer family. Once phosphorylated, AHP enters the nucleus and in turn activates ARR-B (Arabidopsis Response Regulator) through phosphorylation. The phosphorylated ARR-B further regulates the transcription of cytokinin-response genes (Bidon et al., 2020; Rashotte, 2021). As far as cyanobacteria are concerned, the presence of a cytokinin receptor homolog, all2875, is evident in *Nostoc sp.* PCC 7120. This receptor, all2875, is a histidine kinase receptor, containing the CHASE domain, and is known to bind with iP (isopentenyl adenine). Moreover, this receptor binds with trans-zeatin also but with lower affinity (Bidon et al., 2020). However, the majority of the proteins required for cytokinin signaling are not organized in cyanobacteria in a way that allows them to contribute to phytohormone signaling.

Another technique for limiting CK degradation is to suppress the CK oxidase/dehydrogenase gene (CKX), which induces the removal of the prenyl side chain from the adenine moiety of cytokinin (Khandal et al., 2020). Apart from that, various inhibitors of CKX, such as Thidiazuron, CPPU (*N*-(2-chloro-4-pyridyl)-*N*′-phenylurea), TD-K (*N*-furfuryl-*N*′-1,2,3-thiadiazol-5-yl-urea), and INCYDE (2-chloro-6-(3-methoxyphenyl) aminopurine), are used to prevent the irreversible degradation of cytokinin. These inhibitors can activate the cytokinin receptor as anti-senescence properties (van Voorthuizen et al., 2020). INCYDE is a particularly potent CKX inhibitor among them (Prerostova et al., 2020). INCYDE has been used to improve plant tolerance to abiotic and biotic stresses such as the cadmium challenge in *Bulbine natalensis* and *Rumex crispus* as well as the salt challenge in tomato (van Voorthuizen et al., 2021) and V*erticillium longisporum* (Prerostova et al., 2020). In a recent study, an analog of INCYDE, INCYDE-F was used in the barley field for altering the endogenous cytokinin content (Koprna et al., 2020).

## 7 Genetic manipulation of the cytokinin biosynthetic pathway

As the climate is changing, the demand for stress-tolerant crops that can adapt to harsh environmental circumstances like drought and heat stress has to be enhanced (Mubarik et al., 2021). The existing crop enhancement approaches, such as grafting (hybridization), polyploidy, backcross method, clonal selection, and mutation breeding are likely to have reached their limits, genetic engineering is projected to be able to increase the crop output even further (Salonia et al., 2020). As a result, the genetic manipulation of plants, widely used in the United States, is offering solutions to farming challenges in dry and tropical economies that rely largely on agriculture. The resource-poor farmers from countries like China, Brazil, and India account for almost 90% of the genetically modified (GM) crop cultivation. Extensive research studies are being carried out in order to generate transgenic plants with enhanced stress resistance. Hereafter, the present article explains the overview of the genetic manipulation strategies for refinement of the plant’s responses to various abiotic stressors, such as DNA methylation, post-transcriptional modification of mRNA, post-translational modifications, tagging RNA molecules, micro-RNAs, epigenetic modification, and CRISPR-Cas9 editing (Mandal et al., 2022). Furthermore, the synthetic biology technologies and approaches will expand the capabilities and perspectives of genetic engineering initiatives aimed at improving the abiotic stress tolerance in plants (Sargent et al., 2022).

The traditional approach to engineer plants for improved abiotic stress tolerance entails interruption at various stages of the response, from sensors and signaling/regulatory elements (such as kinases, transcription factors) to effectors like osmo-protectants, phytohormones, antioxidant enzymes, and heat-shock proteins (Tanpure et al., 2021). Ramireddy et al. (2018) created transgenic barley plants with an expanded root system by accelerating cytokinin breakdown in the roots by the production of a cytokinin oxidase/dehydrogenase under the control of root-specific rice promoters. They also showed that, using cytokinins to influence root growth and branching resulted in an increase in macro and microelement levels in transgenic barley plants’ leaves and seeds. This was complemented by an increased drought tolerance, demonstrating that root engineering of cereals is an appealing method for avoiding nutrient deficiency in agronomically important plants.

Another tactic for increasing production is to extend the lifespan of plants to delay senescence. For the first time in tobacco transformation, researchers used an *Arabidopsis* senescence-specific promoter (PSAG12) fused with the IPT gene, resulting in better flowering, seed yield, biomass, and reduced leaf and floral senescence (Salvi et al., 2021). SAG promoters such as SAG12 and others have been widely used in plant transformation vectors since then. The Senescence Associated Receptor Protein Kinase (PSARK) promoter, which is induced by stress during maturation, has also been effectively used in the generation of transgenic plants with greater CK content (Salvi et al., 2021). The use of inducible promoters for the conditional expression of CK-biosynthetic genes allows for hormone modulation without the negative consequences of large amounts of this phytohormone on plant growth and development (Salvi et al., 2021).

Recently, it has been observed that the CK biosynthesis pathway in *Nostoc* is activated during the dark period, which results in the gradual increase of CK content once the light phase begins (Frébortová et al., 2017). It was revealed in a transcriptome-based study that the application of cytokinin in the presence of light (CK alone was unsuccessful) changed the transcript levels of numerous genes, such as signal transduction, including two-component sensor histidine kinases and two-component hybrid sensors and regulators in cyanobacteria (*Nostoc* sp. PCC 7120) (Frébortová et al., 2017).

Genetic engineering of cytokinins (CKs), appears to be a viable method for increasing plant productivity, both the synthetic and catabolic routes of phytohormones can be engineered to regulate their levels (Mubarik et al., 2021). Plant yield and productivity have long been thought to be affected by cytokinins, which regulate many aspects of plant growth and development. Genes involved in CK synthesis (*ipt* encoding isopentenyl transferase) and metabolism (CKX encoding cytokinin dehydrogenase and the genes of glucosyltransferases) are the main targets of CK engineering (Mandal et al., 2022).

The applications of nanotechnology for plant transformation have opened new avenues for understanding cytokinin biology. Researchers have demonstrated that tools such as carbon nanotubes and the widely used CRISPR-Cas9 genetic editing might be used to efficiently change the plant’s genetic material (Demirer et al., 2021). A wide variety of crops have been successfully modified using the CRISPR/Cas technique due to its precision, simplicity, and low cost (Mandal et al., 2022; Yan et al., 2022). Nanomaterial-mediated gene editing now provides the benefits of species independence, ease of use, acceptable preservation of external nucleic acids, strong biocompatibility, high transformation efficiency, and the potential for plant regeneration (He et al., 2019). Exogenous genes are delivered into the cytoplasm or organelle by nanomaterials via an endocytic or non-endocytic route. Raspor et al. observed that an overexpression of the AtCKX2 gene from *Arabidopsis thaliana* in *Solanum tuberosum* L. cv. Désirée resulted in considerably fewer bioactive cytokinins than the control (Mandal et al., 2022). More so, researchers discovered that an overexpression of the CKX gene causes CK deficit in *Hordeum vulgare*, which in turn hinders blooming and causes morphological alterations such as larger root systems and delayed aerial development (Mandal et al., 2022). Silencing the TaCKX2.4 gene in *Triticum aestivum* lowered the activity of the cytokinin oxidase in transgenic plants, leading to cytokinin accumulation (Li et al., 2018). Similarly, Tao et al. (2020) reported that CRISPR-edited RGG1 (G proteins) in *O. sativa* lowered endogenous cytokinin levels, grain size, and plant development. Combining rapidly evolving advancements in the CRISPR/Cas tool with knowledge of the IPT or CKX gene function could speed up the development of new, higher-yielding cultivars that could shape future agricultural practices (Jiang et al., 2021).

## 8 Conclusion

In view of a paradigm shift in climate, and extensive use of chemicals, it is indispensable to cultivate stress-tolerant crops, that too in a sustainable way. Cyanobacteria can be used as a preferred source of exogenous cytokinins to enhance the stress-tolerance capacity, growth, and productivity of plants. These organisms when inoculated in the field may form a symbiotic association with plants and increase the cytokinin level in plants. On the other hand, the cyanobacterial filtrate may also be used directly in the field. Moreover, the biomass or the spent media of cyanobacteria may be used as a source of cytokinin in the tissue culture of various explants. As the metabolism of cytokinin in cyanobacteria is well understood, its production may be enhanced using genetic engineering and synthetic biology approaches. Thus, cyanobacteria, as a source of exogenous cytokinins, have the capability to boost the agro-economies in a sustainable way to fulfill the continuously increasing demand of organic agricultural products. However, more research is needed to ensure the cost-effective availability of cyanobacterial biomass at a large scale as well as the easier techniques for detection and quantification of cytokinins in the biomass. More so, the techniques for co-culturing the cyanobacteria with different plant species should be developed to promote the hassle-free application of cyanobacteria in agriculture.
